# *SMAD3* gene rs12901499 polymorphism increased the risk of osteoarthritis

**DOI:** 10.1042/BSR20180380

**Published:** 2018-05-15

**Authors:** Hao-Yu Yang, Wen-Hao Hu, Tao Jiang, Hui Zhao

**Affiliations:** 1 Department of Orthopedics, The Affiliated Changzhou No. 2 People’s Hospital of Nanjing Medical University, Changzhou 213003, China; 2 Department of Orthopaedics, Huaian First People’s Hospital, Nanjing Medical University, Huaian, China; 3 Department of Orthopaedics, Wuxi People’s Hospital Affliated to Nanjing Medical University, Wuxi, Jiangsu, China; 4 Department of General Surgery, Wuxi Third People’s Hospital, Wuxi, China

**Keywords:** meta-analysis, osteoarthritis, rs12901499 polymorphism, SNP, SMAD3

## Abstract

A growing body of evidence suggested that *smad family member 3* gene rs12901499 polymorphism was associated with the risk of osteoarthritis. However, the results of previous studies were conflicting. In the present study, we assessed whether *smad family member 3* gene rs12901499 polymorphism was associated with the risk of osteoarthritis by the meta-analysis. We searched in the databases of PubMed, Embase, and CNKI. Pooled odds ratios and 95% confidence intervals were calculated. Seven papers involving 11 studies (5344 cases and 9080 controls) analyzed the association between *smad family member 3* gene rs12901499 polymorphism and osteoarthritis risk. This meta-analysis confirmed that *smad family member 3* gene rs12901499 polymorphism increased the risk of osteoarthritis. Stratification analysis of ethnicity found that rs12901499 polymorphism increased the risk of osteoarthritis among both Asians and Caucasians [G vs A: Asians, OR and 95%CI, 1.34(1.07, 1.69), *P*=0.012; Caucasians, OR and 95%CI, 1.21(1.13, 1.29), *P*<0.001]. In addition, subgroup analysis by type of osteoarthritis revealed that *smad family member 3* gene rs12901499 polymorphism was correlated with the increased risk of hip osteoarthritis, but not associated with knee osteoarthritis. Sensitivity analysis did not draw different findings. In conclusion, this meta-analysis indicates that *smad family member 3* gene rs12901499 polymorphism increased the risk of osteoarthritis.

## Introduction

Osteoarthritis (OA), a complex and multifactorial disease, is the most common degenerative arthritis characterized by the degeneration of articular cartilage with joint space narrowing, osteophyte formation, and subcondral sclerosis resulting in pain and joint stiffness [1,2]. Accumulating evidence suggests that aging, genetic predisposition, obesity, inflammation, and excessive mechanical loading predispose to OA development [3]. Epidemiological studies suggested that OA has a strong genetic component. A number of candidate genes, such as encoding collagens (particularly for type II collagen) and other structural proteins of the extracellular cartilage matrix, have been deemed to susceptibility loci for primary OA [4,5].

Smad family member 3 (SMAD3) locates on chromosomes 15q21-22. SMAD3 is a key intracellular messenger of the transforming growth factor-β (TGF-β) signaling pathway, which is an important growth factor to the integrity of articular cartilage [6,7]. TGF-β stimulates proteoglycan and type II collagen synthesis, can down-regulate cartilage-degrading enzymes, and is able to counteract interleukin-1-induced suppression of proteoglycan synthesis [8]. Increasing evidence suggests that TGF-β takes important part in the pathogenesis and progression of OA by functioning as key regulators in bone formation, remodeling, and maintenance [6,9,10]. Therefore, it is reasonable to hypothesize that the *SMAD3* may be a candidate gene for OA susceptibility.

Recently, several studies explored the relationship between *SMAD3* gene rs12901499 polymorphism and OA risk [11–17]. However, the results of these studies were conflicting and inconclusive because of the clinical heterogeneity, different ethnic populations, and small sample sizes. To precisely elucidate the genetic role for *SMAD3* gene rs12901499 polymorphism in the development of OA, we performed a comprehensive meta-analysis to clarify the association between this single nucleotide polymorphism (SNP) and OA risk.

## Materials and methods

### Identification of eligible studies and data extraction

We performed a comprehensive literature search throughout PubMed, Embase, and CNKI databases to retrieve the genetic association studies of OA. The following terms were used in our searching strategies: “Smad family member 3”, “SMAD3”, “SNP”, “polymorphism”, “variant”, “osteoarthritis”, “OA” to identify the publications reporting on *SMAD3* gene rs12901499 polymorphism and OA risk. Additional usable data were obtained by hand searching the bibliographies of genetic association studies on the subject in this analysis. We used no restrictions on the number of samples and language to minimize publications bias. All studies were carefully selected and were up to date as of March 1, 2018. The inclusion criteria for studies were as follows: (1) studies that evaluated the association between *SMAD3* gene rs12901499 polymorphism and OA, (2) studied on human beings, and (3) contained genotype data for the calculation of odds ratios (ORs) and 95% confidence intervals (CIs). The following information was extracted from each study: author, year of publication, ethnicity based on the continent of origin of the study population, type of OA, source of controls (SOC), numbers of cases and controls, and the genotype methods.

### Statistical analysis

ORs and 95%CIs were used to evaluate the strength of correlation between *SMAD3* gene rs12901499 polymorphism and OA risk. Stratification analyses were carried out by ethnicity, SOC, type of OA, Hardy–Weinberg equilibrium (HWE), genotype methods, and study quality. *P*<0.05 was considered statistically significant. Multivariate ORs and corresponding 95% CIs between extreme levels of annualized case volume (highest vs lowest) were pooled using a random-effects model, accounting for clinical heterogeneity. Heterogeneity across studies was assessed by using the Q statistic with its *P* value and *I*^2^ statistic [18,19]. Pooled ORs and 95% CIs were calculated in our meta-analysis which was performed using the following genetic models: (1) allele, (2) recessive, (3) homozygous, (4) heterozygous, and (5) dominant. The power of this meta-analysis was calculated with a significant value of 0.05 [20]. Two reviewers independently performed the extraction of data and assessed the study quality according to Newcastle–Ottawa scale (NOS) [21]. All disagreements were discussed and resolved with consensus. We tested HWE in controls by a Pearson’s χ2 test (available in: http://ihg.gsf.de/cgi-bin/hw/hwa1.pl). The data analyses were conducted with Stata 11.0 software (StataCorp, College Station, TX, U.S.A.). Potential publication bias was assessed by Begg’s and Egger’s linear regression test [22]. *P*<0.05 was considered to indicate statistically significant. We performed sensitivity analysis by omitting each study in turn to determine the effect on the test of heterogeneity and evaluated the stability of the overall results.

## Results

### Characteristics of the included studies

**Figure 1:**
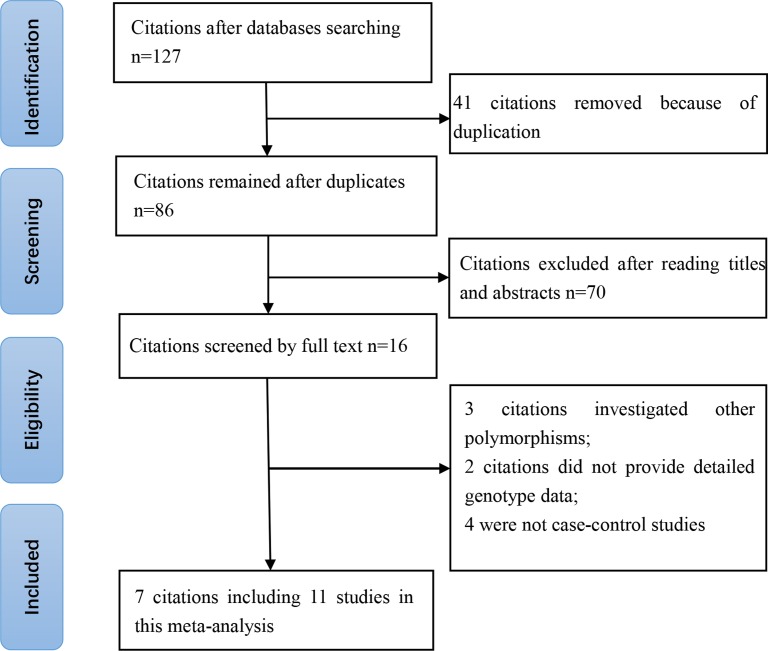
Selection for eligible citations included in this meta-analysis

A total of 127 citations were derived after incipient search. Forty-one citations were removed due to duplication. Of the 86 remaining citations, 70 were excluded after reading titles and abstracts. Sixteen citations were selected for further full-text review. Three investigated other SNPs of *SMAD3* gene; two citations did not provide detailed genotyping data and four were not case–control study. Eventually, we identified seven eligible citations (5344 cases and 9080 controls) containing eleven studies [11–17]. Selection for qualified studies was presented in Figure 1. The characteristics of included studies are summarized in Tables 1 and 2. The NOS of all included studies ranged from 5 to 7 stars, suggesting that these studies were of high methodological quality.

**Table 1:** Characteristics of included studies

Author	Year	Nationality	Type	Number of cases/controls	Genotype method
Sharma, 2017 [11]	2017	India	Knee OA	450/458	PCR­RFLP
Su, 2015 [12]	2015	China	Knee OA	545/468	PCR-RFLP
Xiao, 2015 [13]	2015	China	TMJOA	114/126	PCR
Jiang, 2013 [14]	2013	China	Knee OA	102/220	PCR-RFLP
Jiang, 2013 [14]	2013	China	Hand OA	111/220	PCR-RFLP
Ana, 2010 [15]	2010	U.K.	Knee OA	1936/1253	KASPar chemistry
Ana, 2010 [15]	2010	U.K.	Hip OA	1193/1253	KASPar chemistry
Ana, 2010 [15]	2010	U.K./Estonia	Knee OA	492/1804	KASPar chemistry
Ana, 2010 [15]	2010	U.K./Estonia	Hip OA	95/1804	KASPar chemistry
Zhong, 2018 [16]	2018	China	Hip OA	500/1080	TaqMan
Zhang, 2018 [17]	2018	China	Knee OA	346/394	MALDI-TOF MS

Abbreviations: OA, osteoarthritis; TMJOA, temporomandibular joint osteoarthritis.

**Table 2:** Characteristics of included studies

Author, year	SOC	Ethnicity	Case					Control					NOS	HWE
			AA	GA	GG	G	A	AA	GA	GG	G	A		
Sharma, 2017 [11]	HB	Asians	165	131	154	439	461	158	198	102	402	514	5	NO
Su, 2015 [12]	HB	Asians	142	274	129	532	558	116	228	124	476	460	6	YES
Xiao, 2015 [13]	HB	Asians	31	53	30	113	115	44	67	15	97	155	7	YES
Jiang, 2013 [14]	PB	Asians	22	68	12	92	112	114	83	23	129	311	6	YES
Jiang, 2013 [14]	PB	Asians	25	73	13	99	123	114	83	23	129	311	6	YES
Ana, 2010 [15]	HB	Caucasians	251	682	463	1608	1184	281	625	347	1319	1187	6	YES
Ana, 2010 [15]	HB	Caucasians	219	584	390	1364	1022	281	625	347	1319	1187	6	YES
Ana, 2010 [15]	PB	Caucasians	94	242	156	554	430	421	896	487	1870	1738	6	YES
Ana, 2010 [15]	PB	Caucasians	18	47	30	107	83	421	896	487	1870	1738	6	YES
Zhang, 2018 [16]	PB	Asians	10	200	290	780	220	20	610	450	1510	650	7	YES
Zhang, 2018 [17]	HB	Asians	82	173	91	355	337	81	202	111	424	364	7	YES

### Meta-analysis of *SMAD3* gene rs12901499 polymorphism

**Figure 2:**
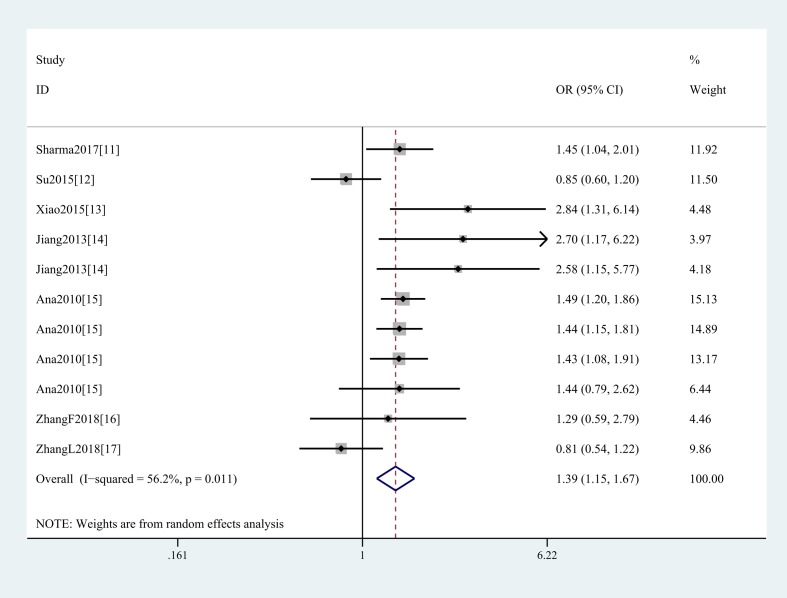
Forest plot shows odds ratio for the associations between rs12901499 polymorphism and OA risk (GG vs AA)

**Figure 3:**
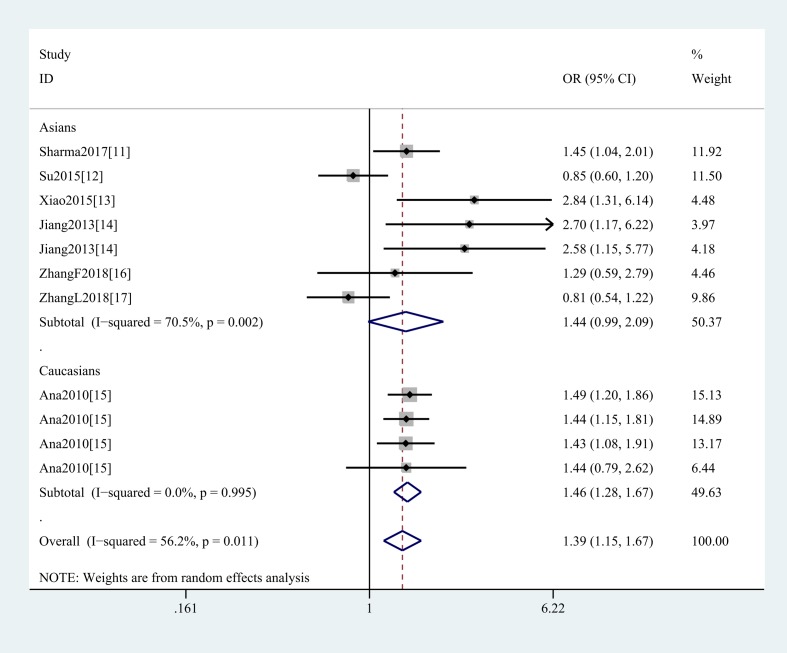
Stratification analysis by ethnicity shows odds ratio for the association between rs12901499 polymorphism and OA risk (GG vs AA)

**Figure 4:**
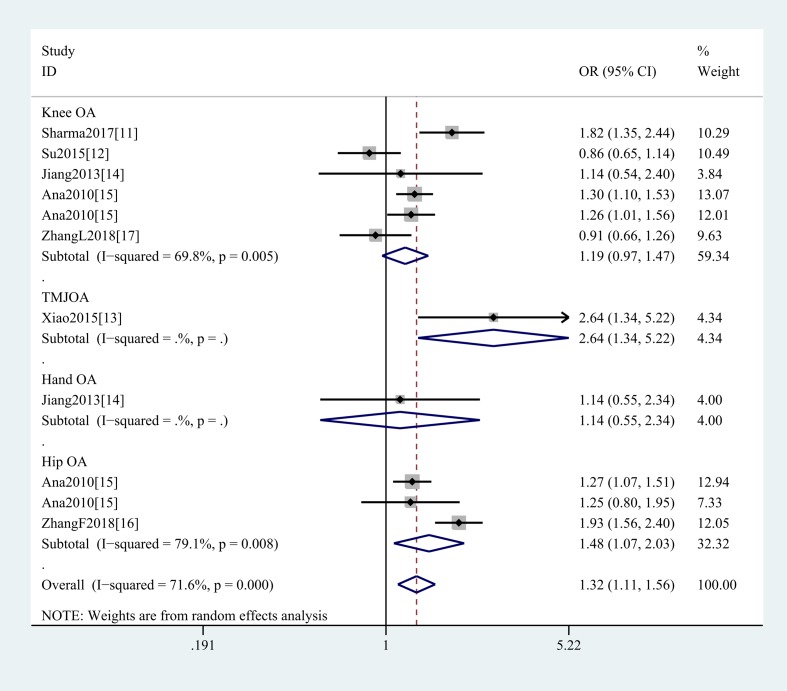
Stratification analysis by type of OA shows odds ratio for the association between rs12901499 polymorphism and OA risk (GG vs GA + AA)

In the general analysis, we found that *SMAD3* gene rs12901499 polymorphism increased OA risk (G vs A: OR and 95%CI, 1.26(1.12, 1.42), *P*<0.001; GG vs AA: OR and 95%CI, 1.39(1.15, 1.67), *P*=0.001; GG + GA vs AA: OR and 95%CI, 1.34(1.07, 1.68), *P*=0.010; GG vs GA+AA: OR and 95%CI, 1.32(1.11, 1.56), *P*=0.001 Table 3 and Figure 2). And we did not obtain any different conclusion after eliminating Su et al.’s study [12] that does not meet the HWE. Stratification analyses were conducted according to ethnicity (G vs A: Asians, OR and 95%CI, 1.34(1.07, 1.69), *P*=0.012; Caucasians, OR and 95%CI, 1.21(1.13, 1.29), *P*<0.001, Figure 3), SOC, type of OA (Figure 4), HWE, genotype methods, and study quality (Table 4).

**Table 3:** Meta-analysis of association between *SMAD3* rs12901499 polymorphism and OA

Comparison	OR (95%CI)	*P*-value	*P* for heterogeneity	*I*^2^ (%)	Model
G vs A	1.26(1.12, 1.42)	<0.001	<0.001	75.8	Random
GG vs AA	1.39(1.15, 1.67)	0.001	0.011	56.2	Random
GG + GA vs AA	1.34(1.07, 1.68)	0.010	<0.001	79.9	Random
GG vs GA + AA	1.32(1.11, 1.56)	0.001	<0.001	71.6	Random
GA vs AA	1.25(0.96, 1.63)	0.101	<0.001	84.0	Random

**Table 4:** Summary of the subgroup analyses in this meta-analysis

Comparison	Category	Category	Studies	OR (95% CI)	*P*-value
G vs A	Ethnicity	**Asians**	**7**	**1.34(1.07, 1.69)**	**0.012**
		**Caucasians**	**4**	**1.21(1.13, 1.29)**	**<0.001**
	SOC	HB	6	1.13(0.99, 1.28)	0.053
		PB	5	1.49(1.22, 1.81)	<0.001
	Type	Knee OA	6	1.16(0.99, 1.35)	0.054
		TMJOA	1	1.57(1.09, 2.26)	0.015
		Hand OA	1	1.94(1.39, 2.71)	<0.001
		**Hip OA**	**3**	**1.30(1.10, 1.55)**	**0.002**
	HWE	Yes	1	1.22(1.01, 1.46)	0.037
		No	10	1.27(1.12, 1.44)	<0.001
	Genotype methods	PCR-RFLP	4	1.40(0.99, 1.98)	0.056
		PCR	1	1.57(1.09, 2.26)	0.015
		KASPar chemistry	4	1.21(1.13, 1.29)	<0.001
		Taqman	1	1.53(1.28, 1.82)	<0.001
		MALDI-TOF MS	1	0.90(0.74, 1.11)	0.335
	Study quality	Medium	8	1.25(1.11, 1.41)	<0.001
		High	3	1.28(0.87, 1.88)	0.025
GG vs AA	Ethnicity	Asians	7	1.44(0.99, 2.09)	0.059
		**Caucasians**	**4**	**1.46(1.28, 1.67)**	**<0.001**
	SOC	HB	6	1.28(0.99, 1.65)	0.055
		PB	5	1.56(1.24, 1.96)	<0.001
	Type	Knee OA	6	1.26(0.98, 1.63)	0.074
		TMJOA	1	2.84(1.31, 6.14)	0.008
		Hand OA	1	2.58(1.15, 5.77)	0.021
		**Hip OA**	**3**	**1.43(1.16, 1.76)**	**0.001**
	HWE	Yes	1	1.45(1.04, 2.01)	0.030
		No	10	1.39(1.12, 1.72)	0.002
	Genotype methods	PCR-RFLP	4	1.53(0.93, 2.54)	0.097
		PCR	1	2.84(1.31, 6.14)	0.008
		KASPar chemistry	4	1.46(1.28, 1.67)	<0.001
		Taqman	1	1.29(0.59, 2.79)	0.520
		MALDI-TOF MS	1	0.81(0.54, 1.22)	0.318
	Study quality	Medium	8	1.42(1.19, 1.68)	<0.001
		High	3	1.36(0.62, 2.88)	0.416
GG + GA vs AA	Ethnicity	Asians	7	1.42(0.91, 2.22)	0.119
		**Caucasians**	**4**	**1.30(1.16, 1.46)**	**<0.001**
	SOC	HB	6	1.10(0.92, 1.30)	0.304
		PB	5	1.88(1.09, 3.25)	0.023
	Type	Knee OA	6	1.23(0.92, 1.64)	0.164
		TMJOA	1	1.44(0.83, 2.49)	0.198
		Hand OA	1	3.70(2.20, 6.21)	<0.001
		**Hip OA**	**3**	**1.26(1.06, 1.51)**	**0.011**
	HWE	Yes	1	0.91(0.69, 1.19)	0.495
		No	10	1.41(1.11, 1.79)	0.005
	Genotype methods	PCR-RFLP	4	1.81(0.89, 3.66)	0.101
		PCR	1	1.44(0.83, 2.49)	0.198
		KASPar chemistry	4	1.30(1.16, 1.46)	<0.001
		Taqman	1	0.92(0.43, 1.99)	0.841
		MALDI-TOF MS	1	0.83(0.59, 1.18)	0.304
	Study quality	Medium	8	1.46(1.12, 1.89)	0.005
		High	3	0.99(0.71, 1.40)	0.975
GG vs GA + AA	Ethnicity	Asians	7	1.37(0.97, 1.92)	0.071
		**Caucasians**	**4**	**1.27(1.15, 1.41)**	**<0.001**
	SOC	HB	6	1.27(1.01, 1.59)	0.037
		PB	5	1.41(1.09, 1.83)	0.010
	Type	Knee OA	6	1.19(0.97, 1.47)	0.102
		TMJOA	1	2.64(1.34, 5.22)	0.005
		Hand OA	1	1.14(0.55, 2.34)	0.729
		**Hip OA**	**3**	**1.48(1.07, 2.03)**	**0.016**
	HWE	Yes	1	1.82(1.35, 2.44)	<0.001
		No	10	1.27(1.07, 1.51)	0.007
	Genotype methods	PCR-RFLP	4	1.21(0.76, 1.90)	0.420
		PCR	1	2.64(1.34, 5.22)	0.005
		KASPar chemistry	4	1.27(1.15, 1.41)	<0.001
		Taqman	1	1.93(1.56, 2.40)	<0.001
		MALDI-TOF MS	1	0.91(0.66, 1.26)	0.568
	Study quality	Medium	8	1.25(1.09, 1.44)	0.001
		High	3	1.60(0.88, 2.92)	0.122
GA vs AA	Ethnicity	Asians	7	1.30(0.76, 2.21)	0.335
		**Caucasians**	**4**	**1.21(1.07, 1.37)**	**0.002**
	SOC	HB	6	0.99(0.80, 1.22)	0.898
		PB	5	1.78(0.94, 3.39)	0.078
	Type	Knee OA	6	1.16(0.82, 1.65)	0.391
		TMJOA	1	1.12(0.63, 2.01)	0.698
		Hand OA	1	4.01(2.35, 6.85)	<0.001
		Hip OA	3	1.14(0.91, 1.44)	0.249
	HWE	Yes	1	0.63(0.46, 0.86)	0.004
		No	10	1.34(1.04, 1.74)	0.025
	Genotype methods	PCR-RFLP	4	1.75(0.73, 4.19)	0.206
		PCR	1	1.12(0.63, 2.01)	0.698
		KASPar chemistry	4	1.21(1.07, 1.37)	0.002
		Taqman	1	0.66(0.30, 1.42)	0.286
		MALDI-TOF MS	1	0.85(0.59, 1.22)	0.373
	Study quality	Medium	8	1.40(1.02, 1.92)	0.038
		High	3	0.88(0.66, 1.17)	0.366

Abbreviations: HB, hospital-based controls; PB, population-based controls; SOC, source of controls; TMJOA, temporomandibular joint osteoarthritis. Medium quality: NOS = 5–6; High quality: NOS = 7.

**Figure 5:**
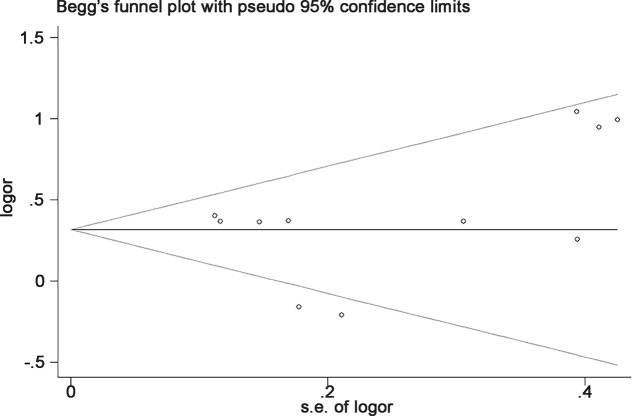
Begg’s tests between rs12901499 polymorphism and OA (GG vs AA)

We assessed sensitivity analysis by omitting each study once at a time in every genetic model for *SMAD3* gene rs12901499. The pooled ORs for the effects of the SNP on the risk for OA indicated that our data were stable and trustworthy. Both Egger’s and Begg’s tests were used to evaluated the publication bias of this meta-analysis. Our data revealed that there was no obvious publication bias for *SMAD3* gene rs12901499 (Figure 5).

## Discussion

To our best knowledge, the present study is the first systematical meta-analysis regarding the association between *SMAD3* gene rs12901499 and OA susceptibility. TGF-β has anabolic effects on chondrocytes especially via the *SMAD3* genes signaling which promote the development and progression of OA [6]. Previous study reported the relationship between the genetic variants of TGF-β itself, TGF-β signaling, and OA [7]. In the signaling pathway of TGF-β, phosphorylated SMAD3 translocates to the nucleus to regulate gene expression and promote an anabolic phenotype in cartilage by forming a complex with SMAD4 [23]. Several previous studies reported the association between *SMAD3* gene rs12901499 polymorphism and risk of OA, but the results were inconsistent [11–17]. This meta-analysis summarized identified seven eligible citations (5344 cases and 9080 controls) containing 11 studies, and provided evidence that *SMAD3* gene rs12901499 polymorphism increased OA risk. Stratification analyses of ethnicity, SOC, type of OA, HWE, genotype methods, and study quality revealed that *SMAD3* gene rs12901499 polymorphism was also correlated with the increased risk of OA.

A single study could be underpowered because of sample size, diversity inheritance of the heterogeneous and complex OA etiology, different ethnicities, clinical heterogeneity, and so on. For instance, Sharma et al. [11] reported an increased association between *SMAD3* gene rs12901499 polymorphism and knee OA in an Indian population. Xiao et al. [13] found this SNP increased the risk of temporomandibular joint OA in a Chinese population. Liying et al. [14] reported this SNP increased both knee and hand OA in a Chinese population. And the study from Valdes et al. [15] indicated that *SMAD3* gene rs12901499 polymorphism is involved in increased risk of both hip and knee OA in European populations. Zhong et al. [16] found that *SMAD3* SNP rs12901499 GA genotype and G variant may increase the risk of hip OA in Chinese Han patients. Zhang et al. [17] confirmed that rs12901499 polymorphism in the *SMAD3* gene plays a protective role in the pathology of knee OA in a Chinese population. However, Su et al. [12] failed to obtain any relationship between *SMAD3* gene rs12901499 polymorphism and knee OA from a Chinese population. It is worthy of note that Valdes et al. [15] conducted eight separate studies (while we divided them into four groups depend on the SOC and type of OA), but three knee OA studies and two hip OA studies did not achieve statistical significance. The nature of OA-genetic susceptibility is likely to vary between different joint sites because the phenotype of osteoarthritis is site-specific. The proportion of genetic contribution of certain polymorphic locus to OA susceptibility may be influenced by other local environmental factors such as anatomical and biomechanical effects and by some joint-specific genetic factors most of which were postulated to be involved in cell signaling and signal transduction. In order to overcome the problem of conflicting results, we performed this comprehensive meta-analysis to evaluate the association of *SMAD3* gene rs12901499 polymorphism with OA risk.

Large sample and unbiased epidemiological studies of predisposition gene polymorphisms could provide insight into the association between candidate genes and diseases. When we dropped the study [11] which is not in agreement with HWE, the increased risk of OA was still found, suggesting the robustness of our findings. In addition, the power analysis indicated that this meta-analysis had a power of 99.9% to detect the effect of rs12901499 polymorphism on OA susceptibility with an OR of 1.26, also indicating that our data were robust. Some limitations encountered in this meta-analysis should be considered when these results are interpreted. First, the heterogeneity of this meta-analysis is high, so the data should be interpreted with caution. Second, due to limited data, we could not conduct further stratification analyses of other potential factors, such as age, gender, and body mass index (BMI). Third, our results were based on unadjusted estimates for confounding factors, which might have affected the final results. Fourth, we could not assess potential gene–gene and gene–environment interactions because of the lack of relevant data. Fifth, the conclusions of some stratification analyses about rs12901499 polymorphism should be interpreted with caution due to limited sample size. Sixth, we only can infer but cannot conclude that *SMAD3* gene rs12901499 polymorphisms are susceptibility loci of other types of OA, highlighting the necessity for the further investigation of more types of OA.

In conclusion, this meta-analysis confirms that *SMAD3* gene rs12901499 polymorphism increased OA risk. Stratification analysis of ethnicity found that rs12901499 polymorphism increased the risk of osteoarthritis among both Asians and Caucasians, subgroup analysis by type of osteoarthritis revealed that *smad family member 3* gene rs12901499 polymorphism was correlated with the increased risk of hip osteoarthritis, but not associated with knee osteoarthritis. Further, studies with large sample size and multiple OA type are necessary to validate whether *SMAD3* gene rs12901499 polymorphism contribute to OA susceptibility.

## Abbreviations

CI: confidence interval
HWE: Hardy-Weinberg equilibrium
NOS: Newcastle–Ottawa Scale
OA: osteoarthritis
OR: odds ratio
SMAD3: SMAD family member 3
SNP: single nucleotide polymorphism
SOC: source of controls
TGF-β: transforming growth factor-β
